# “Please keep on beating”—Participation in a Creative Workshop Offers Unexpected Benefits to Women With Takotsubo Cardiomyopathy

**DOI:** 10.1177/23743735231151765

**Published:** 2023-06-27

**Authors:** Jo Wray, Sofie Layton, Maria Vaccarella, Chiara Bucciarelli-Ducci, Giovanni Biglino

**Affiliations:** 1 Centre for Outcomes and Experience Research in Children's Health, Illness and Disability (ORCHID), 4956 Great Ormond Street Hospital for Children NHS Foundation Trust, London, UK; 2 NIHR Great Ormond Street Hospital Biomedical Research Centre, London, UK; 3 4910 Royal College of Art, London, UK; 4 Department of English, University of Bristol, Bristol, UK; 5 4964 Royal Brompton and Harefield Hospitals, Guys’ and St Thomas’ NHS Trust, London, UK; 6 School of Biomedical Engineering and Imaging Sciences, Faculty of Life Sciences and Medicine, King's College University, London, UK; 7 Bristol Medical School, University of Bristol, Bristol, UK; 8 National Heart and Lung Institute, Imperial College London, London, UK

**Keywords:** Takotsubo cardiomyopathy, creative workshop, experience, unmet need, psychological support

## Abstract

Takotsubo cardiomyopathy (TCM) or “broken heart syndrome” is a rare condition that is more common in women than men, particularly those who are postmenopausal. It mimics a myocardial infarction and psychological factors have been implicated in its etiology as well as being consequences of its presentation. As part of a public engagement project we brought together 8 women (of 12 invited) previously diagnosed with TCM to facilitate a discussion, through participation in a creative workshop-based process, about their illness experience, how they made sense of it, and the meaning it had for them in their lives, and to identify areas of unmet need. Through a range of creative activities we identified that participants had high levels of unmet need in terms of information and psychosocial support. All participants enjoyed the creative process and meeting other people with a diagnosis of TCM. The workshop overall was perceived as empowering. Exploring patient narratives during artist-facilitated workshops is one approach for providing the first steps to addressing unmet need, although the importance of ensuring psychological safety cannot be over-stated.

## Introduction

Takotsubo cardiomyopathy (TCM) or “broken heart syndrome” is a rare condition first described in Japan in the 1990s.^1^ It is a non-ischemic cardiomyopathy induced by the release of catecholamines following an intense negative or positive emotional or physical stress and in 2006 was categorized by the American Heart Association as a form of acquired cardiomyopathy.^2^ TCM mimics a myocardial infarction both in symptoms and electrocardiographic appearance, resulting in a temporary and reversible heart dysfunction with a characteristic ventricular ballooning shape, most typically at the apex.^3^ It is typically much more commonly seen in women than men, with different mechanisms of onset, and is particularly prevalent in postmenopausal women.^4^ Decreased estrogen levels are a pathogenic mechanism of Takotsubo (linking with the high prevalence in postmenopausal women). Other mechanisms have been indicated, including women having a more significant increase in the extracellular matrix-receptor interaction than males and different pathological findings after hematoxylin–eosin staining in males and females. TCM is usually reversible but can be associated with clinical complications such as heart failure and, on rare occasions, malignant arrhythmia and fatal complications such as left ventricular free wall rupture.^5^ Recurrence has been reported in 5% of cases.^6^ As stated in 2015, “the natural history, management, and outcome of Takotsubo (stress) cardiomyopathy are incompletely understood.”^7^

Psychological factors are implicated in many forms of heart disease, including TCM, and there is evidence that anxiety, depression, posttraumatic stress, and some personality traits are associated with TCM.^8–10^ Preexisting psychiatric illness has also been found to be associated with an increased risk of recurrent TCM.^11^ In a narrative interview study with 19 people diagnosed with TCM, the salience of long-term stressful circumstances resulting in vulnerability to acute psychological or physical stressors and the subsequent onset of TCM was identified.^12^ Participants reported residual symptoms of pain, sleep disturbance, and fatigue 8 weeks after hospital discharge following treatment for TCM^13^ as well as feeling “alone and lost” in terms of their symptom burden, which had a resulting impact on their health and ability to return to daily life.^14^ Although there is a growing evidence base about the precursors to, and psychological concomitants of, TCM, less is known about meanings ascribed by patients to TCM, or about their psychological needs and if and how these are addressed following a diagnosis of TCM. More recently, since the inception of the COVID-19 pandemic, an increased incidence of TCM has been reported in both the general population and patients with COVID-19, suggesting a significant and likely under-reported impact which may be related to the virus itself and/or a response to the adverse effects of COVID-19 on mental health.^15^

One challenge in furthering our knowledge about longer-term outcomes of people in the United Kingdom previously diagnosed with TCM is that they are often discharged from all follow-up with cardiology services. Our aim, therefore, as part of a public engagement project, was to bring together a group of women previously diagnosed with TCM but not under follow-up in order to facilitate a discussion through participation in a creative workshop-based process about their illness experience, how they made sense of it and the meaning it had for them in their lives and, in so doing, identify areas of unmet need.

## Methods

Women previously diagnosed with TCM in a tertiary specialist cardiology service were invited to attend a six-hour workshop in July 2018. Written information about what would be involved was sent to potential participants and they were asked to contact the team if they were interested in attending the workshop. Inclusion criteria were a definitive diagnosis of TCM and age < 75 years. The workshop was held in a non-hospital setting and was facilitated by an artist, health psychologist, bioengineer, lecturer in medical humanities, and cardiologist. Refreshments were provided and travel expenses were offered. The format for the day Table 1 followed an approach previously used by our team with other cardiac patients in earlier public engagement work.^16^

**Table 1.** Workshop Process.

1. Written informed consent and “housekeeping” 2. Introductions + 3 words 3. Blind self-portraiture (2-dimensional—drawing) 4. Blind self-portraiture (3-dimensional—sculpting) 5. Brief reflection 6. Creative writing exercise—if you were a …, what … would you be and why • Animal • Color • Vegetable • Weather/element • Piece of furniture • Book/genre of book • Building 7. Body mapping exercise • Contouring in pairs • Filling in the body map 8. Discussion of body maps 9. Group reflection 10. Letter to your heart 11. Conversation with a cardiologist (CB-D) 12. 3 words

Participants provided written consent for the workshop to be recorded and for their artistic outputs to be photographed and anonymized quotes used in dissemination. Prior to the workshop starting they were asked to write down 3 words to reflect how they were feeling. Each participant was given the opportunity to briefly “tell their story” before beginning the creative activities. At the end of the workshop participants were invited to write a letter to their heart, which formed the basis of the reflective session along with their body maps. Those who felt able to were individually invited to share their letter with the rest of the group. Particular attention was given to ensuring that all participants had an opportunity to share their views and that individual participants did not dominate the discussion. The psychologist (JW), artist (SL), and bioengineer (GB) facilitated the discussion, all of whom are experienced in workshop facilitation and have worked together in similar situations previously. Participants were also asked to write down 3 words about how they felt about having completed the workshop. During this final session they had the opportunity to ask the cardiologist (CB-D) questions about TCM and in turn, were asked about what would be helpful for them and other patients with TCM in the future. Contemporaneous notes were made during the workshop by the psychologist (JW) to capture nonverbal communication and interactions and MV observed the whole workshop, focusing particularly on the language used by participants.

### Analysis

The whole recording was listened to and sections when participants were invited to “tell their story” and the feedback session at the end of the workshop were transcribed. Transcripts and letters to the heart were analyzed thematically, following the staged approach of Braun and Clarke.^17,18^ One author (JW) read the transcripts, letters, and notes, before attaching codes to segments of data and bringing similar codes together to generate themes. The co-authors, all of whom had been present at the workshop, subsequently read the sources of material and generated themes to determine if the proposed themes were accurate representations of the data.

### Ethical Considerations

Formal ethical approval was not required as this was a public engagement project and not clinical research according to the NHS Health Research Authority^19^ but ethical principles were adhered to throughout and underpinned all aspects of the work. Participants provided written informed consent and were made aware that they could leave the workshop at any stage. Particular attention was given to ensuring their psychological safety, which was important given the sensitive nature of the topic and potential antecedents to TCM presentation.

## Results

**Figure 1.**
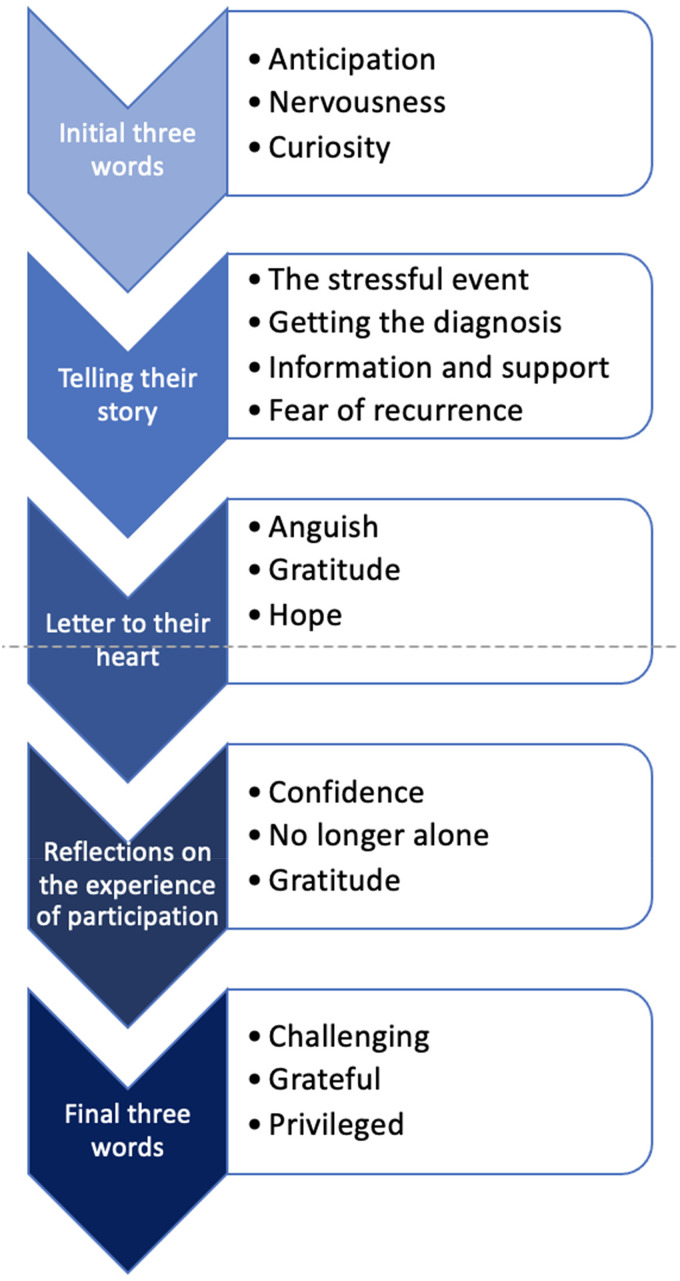
Themes generated from phases of the workshop.

Themes from each phase of the workshop are presented Figure 1, together with anonymized illustrative quotes, with participant numbers shown in brackets.

Twelve women were invited to attend the workshop, 8 (aged 48-75 years) of whom agreed and attended.

### Initial 3 Words

**Figure 2.**
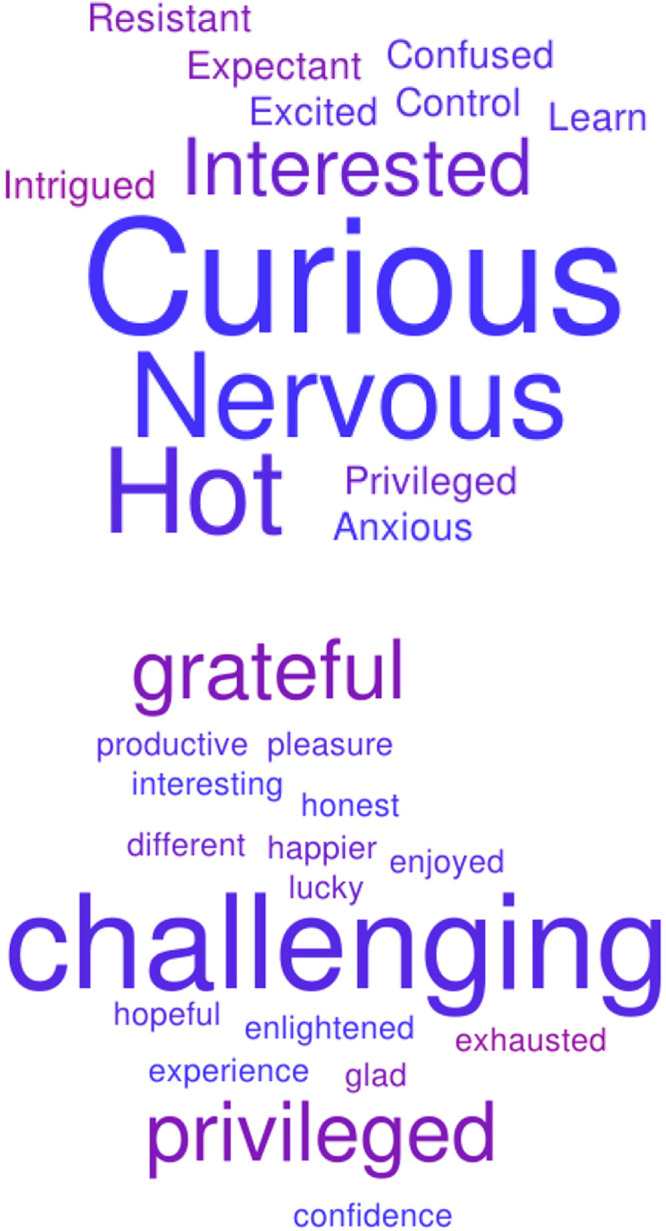
Word clouds illustrating the 3 words at the beginning (top) and end (bottom) of the workshop.

At the start of the workshop, there was a general feeling of anticipation, curiosity, and nervousness, illustrated by the 3 words provided by participants Figure 2. One participant wrote that she was “really pleased to be offered an opportunity to think about my experience differently because mostly it brings anxious thoughts and difficult feelings” (P1).

### Telling Their Story

During the expression of their own individual narratives, themes related to the stressful event itself, getting the diagnosis, the need for (but often lack of) information and support, and a fear of recurrence were articulated. All participants were willing to share elements of their personal stories, which were characterized by a range of individual stressful events preceding the onset of TCM, ranging from close family bereavement to disputes with neighbors, with one participant identifying a physical rather than an emotional stressor. Despite commonalities in their stories, each experience was clearly unique. They discussed their anxieties and fear at the onset of symptoms—“I needed help to know how to cope with the stress” (P4)—the difficulties around the diagnosis and associated uncertainty and sense of ignorance about what might happen, and their challenges with accessing information and support. One participant described how she was “concerned about any permanent damage that doesn’t show up … it is all very worrying … I feel if I knew a bit more … you can accept it more if you know the truth” (P2). There was a strong sense of the isolation they felt, in terms of others not appreciating what TCM was—“My heart just causes me worry…something I don’t share with people” (P1)—or not knowing others who had had similar experiences, which increased their feelings of vulnerability. They also talked about their anxiety that they might get TCM again, particularly because of other health conditions requiring treatment, “I know I have to go for hip surgery, I am putting it off … in case I have Takotsubo again … I’m scared” (P3).

### Creative Activities

Initial engagement with the activities varied—some participants became completely immersed while others were more reserved, taking longer to settle and join in with the group work. During the activities participants expressed a range of emotions—one became angry and upset and temporarily left the group, and others became tearful. In contrast, during the body mapping exercise participants chatted together about their experiences and clearly enjoyed the opportunity to share their stories with others who had been given the same diagnosis of TCM.

### Letter to Their Heart

Participants’ letters (range: 20-117 words) to their hearts revealed themes related to anguish, gratitude, and hope, and for some, the experience of writing and sharing was particularly emotional, “It brings such memories for me” (P7). One participant also wrote of her seemingly conflicting feelings about her heart, describing it as, “Friend and foe” (P8). The theme of anguish was particularly evident—several participants wrote of their desperation for their heart to “please keep beating” (P7), which was linked to their future wishes, “keep going, I have a lot of things I still want to do” (P6). Another participant wrote, “Please don’t stop beating, I love my family too much” (P1) while another expressed, “I wish you would beat without me hearing or feeling you every minute of [the] day and night—I wish you could become happy again so I could laugh and smile” (P5). They also described a sense of gratitude, “I want to thank you for working and trying so hard to restore yourself after your left ventricle collapsed—through no fault of your own” (P2). The letters portrayed a sense of hope for the future, “I sing for joy … hope this helps you as well as me” (P6) and “My time is not over and I feel you will not let me down” (P7), with some “bargaining” with their heart, “I promise to eat a healthy diet, take lots of exercise and we will all work together and have a wonderful life for many, many years” (P4). Interestingly, the terminology related to TCM and “broken heart” syndrome also evoked a response, with one participant writing, “You didn’t break so I don’t like ‘Broken Heart’” (P3) while another wrote, “I don’t want to romanticise you like a lobster pot^1^ or a Broken Heart … I prefer your dysfunction to have a medical name and recognition of your importance and value” (P2) whereas another commented, “Takotsubo—how exotic for what some would call a fake heart attack” (P8).

### Reflections on the Experience of Participation

**Figure 3.**
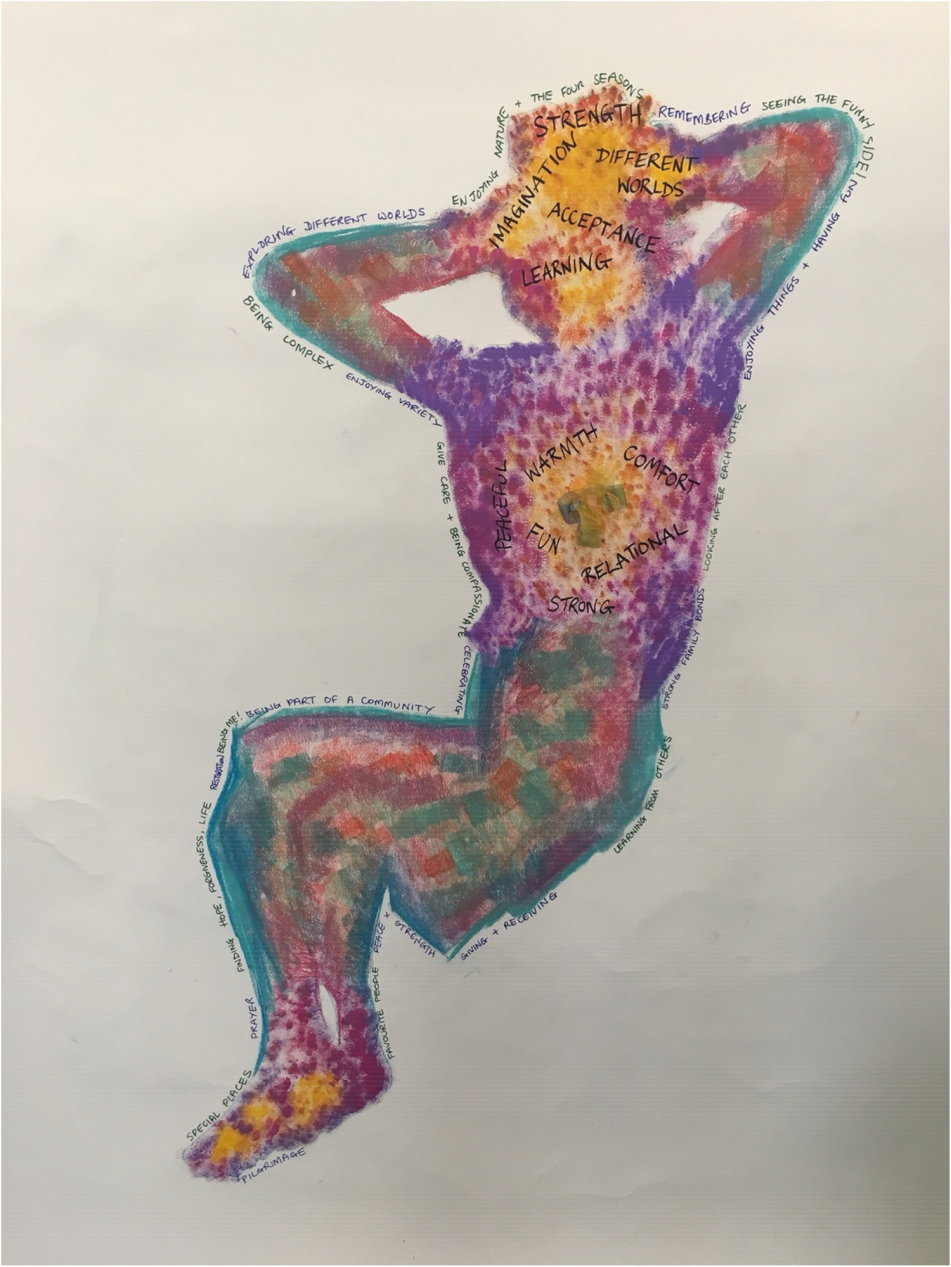
Example of a body map. The outline of the body was drawn in ink and filled in with pastels.

During the final reflection, which lasted approximately 80 min and was structured by a discussion of the body maps (an example is shown in Figure 3), letters, and 3 words (Figure 2), themes related to confidence, no longer feeling alone and gratitude were expressed. Participants talked about the workshop giving them confidence and of enjoying it—“To surrender myself to this day has been magic” (P2)—and the benefits from meeting other people who had been through similar experiences. Being able to voice and share their stories resulted in them feeling less alone, and they expressed a sense of gratitude and privilege and, for some, a realization that others had had worse experiences than they had. One participant commented, “I feel privileged to be here, happier that I have met other people who have the same condition … not feeling so alone” (P3) while another said, “[Today] has given me confidence … I am so glad to have been invited” (P5). Although the sense was of positivity, articulating those experiences and emotions, often for the first time, had also been challenging.

The discussion (∼40 min) with the cardiologist focused on the clinical elements of TCM in terms of causes, symptoms, and treatments and the need for information, support, and underlying precipitating issues to be addressed. The lack of follow-up and psychological support following a diagnosis of TCM, given the emotional stress-induced presentation in many cases, was identified as an area that particularly needed prioritizing. Participants made suggestions about how services and support could be improved, including involving patients in research to ensure their voice is heard:

- Provision of “generic” written information covering causes of TCM, symptoms, treatment, and recurrence risk (in accessible formats and language)
- Patient-specific information, including prognosis
- Psychological support from the time of diagnosis through to postdischarge and beyond
- Normalization of experiences
- Opportunities to “creatively” express feelings
- Lifestyle advice
- Individual peer support
- TCM support group
- TCM website—to include trusted information, digital patient stories, latest research (with lay summaries and links to publications), signposting to other TCM resources
- Involvement in research—patient and public involvement and engagement; participating as co-researchers

Participants were grateful to have been given the chance to talk to a cardiologist and find out more about TCM, highlighting the limited opportunities for discussion during their hospital stay. It also emerged that the change of diagnosis from myocardial infarction (diagnosis at admission) to a non-myocardial infarction (TCM) at discharge created a degree of confusion and uncertainty. The team, in turn, were able to reflect on the emotional rollercoaster these former patients had been on (and for some, were on still) and what an eventful and impactive journey it had been for them. The team valued hearing participants’ stories and appreciated the honesty and generosity of sharing experiences.

### Final 3 Words

At the end of the workshop participants’ 3 words suggested that although they had found aspects of the experience challenging, they were also grateful and felt privileged to have been able to take part (Figure 2).

## Discussion

The combination of the uncommon diagnosis of TCM, the incomplete understanding of its etiology but the recognized association with physical and emotional stressors, the lack of easily accessible patient information, and the dearth of standardized follow-up (particularly psychological support) suggests that affected patients are at the center of a perfect storm. Through a public engagement project, we endeavored to understand the perspectives of a small group of women who had been diagnosed with TCM but were not receiving any follow-up and to ascertain their needs in relation to their diagnosis.

None of the women attending the workshop had previously met anyone else who had had TCM and they all described feelings of anxiety related to their condition, lack of knowledge, and uncertainty about what the diagnosis meant for them and whether it might reoccur, as described previously.^8–14^ The workshop provided them with an opportunity to meet others, thereby reducing their feelings of isolation, and articulate feelings about TCM, corroborating findings about the value of peer support and sharing of experiences in other health conditions.^20,21^ It also provided an opportunity to explore and honor women's individual stories, a concept which is widely acknowledged as not only beneficial but fundamental to the holistic care of patients.^22^ Participants reported feeling more confident and empowered having attended the workshop, which was a benefit that they had not anticipated prior to attending. This was further evidenced by an email received from one participant a few weeks later, in which she described now having the confidence to take on new challenges as a result of sharing her experiences with peers and professionals during the workshop.

There was good engagement with all of the creative activities, with provisions made as necessary to accommodate different physical needs with body mapping in particular. All participants chose some imagery that represented strength and resilience—such as an elephant, “because they have strong family bonds”—or peace—reflected in the choice of weather and colors. Writing letters to their heart, although emotionally challenging, enabled the articulation of feelings and self-expression and helped participants to make sense of their experience. Such an approach has been used with other groups who have experienced trauma—for example, war veterans who experienced moral injury, to help them “contact their emotions”^23^ and the opportunity to engage in creative and writing exercises was one suggestion from the group about what should/could be offered to people diagnosed with TCM, although the need to ensure participants’ psychological safety was also highlighted.

What was particularly striking was the lack of psychological support offered to any of the women. While there are recommendations for monitoring and treatment of physical symptoms of TCM,^24^ and increasing recognition of the role that psychological stressors play in the etiology of TCM,^25,26^ there are no guidelines of which we are aware for provision of psychological support for this patient group, which is a surprising gap. Participants clearly articulated their need for support to help them deal with the stressor that precipitated the onset of TCM but also to manage their anxieties about TCM itself. They identified strategies that would be helpful, primarily about the development and provision of resources to inform and support patients, and these ideas were endorsed by the professionals. The workshop format and the presence of a cardiologist, while enabling participants to ask and get answers to questions, learn more about TCM, and have some misperceptions and fears about TCM allayed, also provided an opportunity for professionals to hear those patient stories, recognized as an important element in providing person-centered, holistic care.^22^

The workshop took place prior to the COVID-19 global pandemic. In a recent review addressing the incidence of TCM within a COVID-19 landscape,^15^ an increase in TCM for populations with and without COVID-19 was identified, suggesting the virus itself was directly linked but also that psychosocial factors such as anxiety and social isolation during the pandemic were drivers for the increased incidence of TCM. Furthermore, the authors suggested there was the potential for seeing further increases in the incidence of TCM associated with future disasters. Our experience with undertaking the workshop indicates a high level of unmet need in terms of coping with TCM and its diagnosis but also offers some solutions. What is now needed is research to identify, implement and evaluate interventions to meet the needs of those diagnosed with TCM, alongside the development of co-created information resources, and we suggest that participatory activities as we have described may be one valuable approach.

It is important to acknowledge the limitations of this public engagement project in terms of the information that we gathered. We did not undertake in-depth interviews or have a topic guide for eliciting responses, so although we collected feedback from participants some of the transcript data are thinner than would be expected via other more traditional means. The group of 8 participants is not necessarily representative of other TCM patients but what the workshop has clearly shown are the benefits of our approach in terms of using creative activities as a means of expression, breaking down barriers, and bringing together people who share commonalities in their experiences.

## Conclusion

While uncommon, TCM is a condition that is increasingly being recognized due to the development of new diagnostic imaging tools. Psychological factors play a key role and our experience with a small group of women previously diagnosed with TCM demonstrates high levels of unmet need in terms of information and psychosocial support. Exploring patient narratives during artist-facilitated workshops offers one approach as a first stage towards addressing this need, although the importance of ensuring psychological safety cannot be over-stated.

## Notes

1. The patient is referring to the origin of the Japanese term for her condition (although she misidentifies the pot—it's used to trap octopuses, not lobsters).
