# Corrigendum to “Please keep on beating”—Participation in a Creative Workshop Offers Unexpected Benefits to Women With Takotsubo Cardiomyopathy

**DOI:** 10.1177/23743735241263462

**Published:** 2024-07-23

Wray J, Layton S, Vaccarella M, Bucciarelli-Ducci C, Biglino G. “Please keep on beating”—Participation in a Creative Workshop Offers Unexpected Benefits to Women With Takotsubo Cardiomyopathy. *Journal of Patient Experience*. 2023;10. doi:10.1177/23743735231151765

To comply with the Open Access policies, grant numbers have been added in the funding section. The corrected funding section is as:

Funding

The author(s) disclosed receipt of the following financial support for the research, authorship, and/or publication of this article: This work was supported by the Brigstow Institute (University of Bristol). The authors also acknowledge support from the Wellcome Trust (201960/Z/16/Z) and from the British Heart Foundation (CH/17/1/32804).

The article is updated online.

